# Supplementary material for: Prevalence of sarcopenia was higher in women than in men: a cross-sectional study from a rural area in eastern China
Source: PeerJ. 2022 Aug 2;10:e13678. doi: 10.7717/peerj.13678 (PMC9354735; doi:10.7717/peerj.13678)
Supplement: Supplemental Information 2 [file peerj-10-13678-s002.pdf]

国家重点研发计划

（项目编号：2018YFC2000301）

我国人群增龄过程中健康状态变化特点与规律研究
基线调查表

ID1 入组标准：是否符合入组标准 □ 是=1 □ 否=2 □ **ID1**

1）在当地具有相对稳定工作与居所的适龄本地居民；

2）无急慢性传染性疾病； 3）无进展性的致命性疾病；

4）无精神疾患； 5）无酗酒或滥用药物史；

6）无犯罪记录；

ID2 排除标准：是否符合排除标准 □ 是=1 □ 否=2 □ **ID2**

1） 一切具有迁徙、调动可能风险的人员。

2） 存在任何躯体或精神功能问题不能完成本调查者

ID3 调查地点 ______省(市) _______市 ______县 □ **ID3**

ID4 本次调查编号: □□□□□□□□□□ □ **ID4**

ID5 调查日期:DATE □□□□/□□/□□ □ **ID5**

ID6 受访者来源： 1=城市社区 2= 城市单位 3= 城镇 □ **ID6**

4=农村 5=医院（体检中心、门诊） 6=其他

ID7 本次问卷调查员编码：□□□□ □ **ID7**

2019 年 3 月

A 基本情况

A1 姓名:______________________ □**A1**

A2 性别：1=男　 2=女 □**A2**

A3 民族：1=汉　 2=蒙　 3=回　 4=藏　 5=其他__________ **A3oth** □**A3**

A4 年龄： _________ 岁 □□□**A4**

A5 完成的最高教育： □**A5**

1=文盲　2=小学　3=初中 4=高中/中专 5=大专　6=本科 7=硕士研究生 8=博士研究生

A6 婚姻状况：

1=未婚　2=已婚同住　3=已婚分居　4=离异　5=丧偶 □**A6**

A7 您现在的工作状况？ □**A7**

1=全职工作　 2=兼职工作　3=不固定（打零工）　4=离/退休　 5=无工作 （跳问 A9）

6=在学（跳问 A9）　 7=其他_____A7oth（跳问 A9）

A8 您目前工作或退休前及曾经的工作岗位是： □**A8**

1=国家机关、党群组织、国有企业、事业单位负责人

2=非国有机构、组织、企业负责人

3=国有单位专业技术人员（科研、工程技术、医疗、经济、法律、教学、文学艺术、体育、媒体）

4=非国有单位专业技术人员（科研、工程技术、医疗、经济、法律、教学、文学艺术、体育、媒体）

5=国有单位办事人员、服务和有关人员（行政办公、安保、消防、邮政、电信、销售、仓储、餐饮、娱乐、运输、医疗辅助、社会服务）

6=非国有办事人员、服务和有关人员（行政办公、安保、消防、邮政、电信、销售、仓储、餐饮、娱乐、运输、医疗辅助、社会服务）

7=国有单位农、林、牧、渔、水利业生产人员

8=非国有单位农、林、牧、渔、水利业生产人员

9=国有单位生产运输设备操作人员（生产工人、工程施工人员、运输设备操作人员、环境监测与废物处理人员、检验、计量人员）

10=非国有单位生产运输设备操作人员（生产工人、工程施工人员、运输设备操作人员、环境监测与废物处理人员、检验、计量人员）

11=军人　12=其他___________________A8oth

A8.1 退休后是否再就业？1=是　　2=否(跳问 A9)　　□ A8.1

A8.2 再就业的工作岗位是：　　□ A8.2

1=国家机关、党群组织、国有企业、事业单位负责人

2=非国有机构、组织、企业负责人

3=国有单位专业技术人员（科研、工程技术、医疗、经济、法律、教学、文学艺术、体育、媒体）

4=非国有单位专业技术人员（科研、工程技术、医疗、经济、法律、教学、文学艺术、体育、媒体）

5=国有单位办事人员、服务和有关人员（行政办公、安保、消防、邮政、电信、销售、仓储、餐饮、娱乐、运输、医疗辅助、社会服务）

6=非国有办事人员、服务和有关人员（行政办公、安保、消防、邮政、电信、销售、仓储、餐饮、娱乐、运输、医疗辅助、社会服务）

7=国有单位农、林、牧、渔、水利业生产人员

8=非国有单位农、林、牧、渔、水利业生产人员

9=国有单位生产运输设备操作人员（生产工人、工程施工人员、运输设备操作人员、环境监测与废物处理人员、检验、计量人员）

10=非国有单位生产运输设备操作人员（生产工人、工程施工人员、运输设备操作人员、环境监测与废物处理人员、检验、计量人员）

11=军人　12=其他______________A8.2oth

A9 现居住住址：　______________ 省（自治区、直辖市）**A9**a　______________ 市（地区、州、盟）**A9**b ______________区（县、市、旗）**A9**c ______________ 乡（镇、街道）**A9**d__________________________街、路、巷、胡同、里 **A9e**

__________________________小区 **A9**f__________________________门牌号 **A9**g

A10 本人联系方式: 手机_________________ **A10**a　家庭电话______________ **A10**b

联系人姓名________________ **A10**c　电话/手机________________ **A10**d

A11 身份证号码：□□□ □□□ □□□□ □□□□□□□□ **A11**

A12 信息来源：　　□ **A12**

1=本人　2=家属　3=保姆　4=其他人_____________ **A12oth**

B 生活方式

B1 您对自己目前的生活满意吗？ □**B1**

非常不满意 非常满意

1	2	3	4	5	6	7	8	9	10

B2 您每周工作 _____天 **B2a**，每天工作约______小时 **B2b**。 □**B2a**□ □**B2b**

B3 您每天用于上班路上的时间（往返），约_______小时。 □**B3**

B4 您每周加班约________小时。 □ □**B4**

B5 请选择自己目前的工作方式? □**B5**

1=脑力劳动为主 2=体力劳动为主 3=脑体结合劳动 4=不清楚

B6 请对自己总体工作状态进行评价： □**B6**

1=我喜欢自己的工作，没有感觉到倦怠。

2=有时感到有压力，不像过去那样有精力，但并没有感觉到倦怠。

3=的确感到倦怠，比如体力或者情绪上的枯竭。

4=感受到的工作倦怠挥之不去，常常在工作中感到沮丧。

5=感到精疲力竭，常常怀疑能否继续下去。需要改变或者寻求帮助。

B7 您每天做家务（包括照看孩子、老人）的时间约_______小时。 □ □**B7**

B8 您每天休闲的时间，约_____小时。 □ □**B8**

B9 在您闲暇时主要做什么？（多选） □ □**B9**

1=上网 2=逛街购物 3=旅游 4=看电影、演出/比赛 5=聊天聚会

6=棋牌 7=锻炼 8=看书/学习 9=其他_____________________**B9oth**

B10 您每天晚上大约_______点上床休息 **B10a**，睡眠时间约_______小时 **B10b**。

□ □**B10a** □ □**B10b**

B11 您是否需要服用安眠药？ 1=从不 2=经常 3=偶尔 □**B11**

B12 您是否可以做到有规律地吃饭、睡觉、排便？ 1=是 2=否 □**B12**

B13 您是否经常参加体育锻炼？ □**B13**

1=否(跳至 B14) 2=是，请回答以下问题（可多选）

方式	种类举例	次/周	分钟/次
B13.1 有氧运动 □	1=走路 2=跑步 3=游泳 4=广场舞	□□B13.1a	□□□B13. 1b
B13.2 平衡类运动 □	1=太极拳 2=________	□□B13.2a	□□□B13. 2b
B13.3 力量型运动 □	1=器械 2=_______	□□B13.3a	□□□B13. 3b
B13.4 柔韧类运动 □	1=瑜伽 2=________	□□B13.4a	□□□B13. 4b
B13.5 球类运动 □	1=乒乓球 2=羽毛球 3=________	□□B13.5a	□□□B13. 5b

B14 您每月为自己的健康保健（保健品、按摩、美容、健身等）花费大约多少元钱？ □**B14**

1=大于 5000 元 2=3001-5000 3=1501-3000 元 4=500-1500 元

5=500 元以下 6=不花费

B15 您的饮食习惯

B15.1 主食习惯： □**B15.1**

1=白米为主 ____两/天 B15.1a 2=面食为主 ____两/天 B15.1b

3=粗粮为主 ____两/天 B15.1c 4=米面兼有 ____两/天 B15.1d

B15.2 食肉习惯： 1=不吃 2=偶尔吃 3=经常吃 4=天天吃 □**B15.2**

B15.3 食蛋习惯： 1=不吃 2=偶尔吃 3=经常吃 4=天天吃 □**B15.3**

B15.4 食鱼习惯： 1=不吃 2=偶尔吃 3=经常吃 4=天天吃 □**B15.4**

B15.5 豆类食品： 1=不吃 2=偶尔吃 3=经常吃 4=天天吃 □**B15.5**

B15.6 奶或奶制品：1=不吃 2=偶尔吃 3=经常吃 4=天天吃 □**B15.6**

B15.7 吃蔬菜习惯：1=不吃 2=偶尔吃 3=经常吃 4=天天吃 □**B15.7**

B15.8 吃水果习惯：1=不吃 2=偶尔吃 3=经常吃 4=天天吃 □**B15.8**

B15.9 饮水习惯： 1=白水 2=喝咖啡为主 3=喝饮料为主 4=喝茶为主 □**B15.9**

B15.10 平均每天饮水多少杯（220ml 一杯，含水、果汁、茶等）？ □**B15.10**

1=至少 3 杯 2=3-5 杯 3=5 杯以上

B16 您是否服用保健品？ □**B16**

1=从不服用　　2=偶尔服用　　3=经常服用

B17 您是否喝酒？ □**B17**

1=不喝酒　　2=现在经常喝酒　　3=过去经常喝，已戒

B18 您是否吸烟？ □**B18**

1=从不吸烟　2=现在吸烟(>5 支/天)　3=偶尔吸烟　4=过去吸，已戒(不吸烟1个月以上)

B19 您既往工作或居住场所是否有人吸烟？ □**B19**

1=没有　2=工作有　3=居住有　4=都有

B20 您现在是否养宠物？　1=否　　2=是 □**B20**

B21 您现在是否感觉到有生活压力？　1=是　　2=否（跳问 B22） □**B21**

B21.1 主要的压力来源是什么？（多选） □**B21.1**

1=个人发展　　2=经济　　3=个人情感　　4=家庭　　5=纠纷、官司

6=人际关系　　7=健康　　8=其他____________B21.1oth

B22 您参加了下列社会组织与活动吗？

组织类型	请选择以下选项			
B22.1 专业技术组织	1 积极成员	2 一般成员	3 未参加	□B22.1
B22.2 运动/娱乐组织	1 积极成员	2 一般成员	3 未参加	□B22.2
B22.3 志愿公益服务组织	1 积极成员	2 一般成员	3 未参加	□B22.3
B22.4 其他组织	1 积极成员	2 一般成员	3 未参加	□B22.4

B23 您是否有宗教信仰？　1=是　2=否 □**B23**

C 身体情况

C1 您对自己的健康情况满意吗？ □**C1**

非常不满意 非常满意

1	2	3	4	5	6	7	8	9	10

请描述您近一年来的身体情况：

C2 您的视力有减退吗？ □**C2**

1=没有 2=有，但不影响日常生活 3=有减退，且影响日常生活

C3 您现在是否佩戴眼镜？ 1=否 2=近视镜 3=花镜 4=近视镜+花镜 □**C3**

C4 您的听力有减退吗？ □**C4**

1=没有 2=有，但不影响日常生活 3=有减退,且影响日常生活

C5 您的牙齿有缺失吗？ □**C5**

1=没有 2=有，但不影响日常生活 3=有脱落，且影响日常生活

C6 您是否觉得自己的记忆力有所下降？ □**C6**

1=没有 2=下降，但不影响日常生活 3=下降，且影响日常生活

C7 您的食欲如何？ 1=减退 2=一般 3=好 □**C7**

C8 您是否有排便困难的情况？ 1=否 2=是 □**C8**

C9 您是否有大便失禁？ 1=没有 2=偶尔 3=经常 □**C9**

C10 您是否有尿失禁？ 1=没有 2=偶尔 3=经常 □**C10**

C11 您现有哪些主要不适？ 1=没有(跳问C12) 2=有 如有请点以下选项 □**C11no**

C11.1 乏力 □C11.1	C11.2 疼痛 □C11.2	C11.3 过敏 □C11.3
C11.4 眼部疲劳 □C11.4	C11.5 情感低落 □C11.5	C11.6 冲动 □C11.6
C11.7 烦躁 □C11.7	C11.8 注意力不集中□C11.8	C11.9 其它__________C11.9oth

C12 请选择以下符合对您个人特征的描写及符合的程度。

	完全不符合	比较不符合	有点不符合	说不清	有点符合	比较符合	完全符合	
E1.1 外向的，热情的	①	②	③	④	⑤	⑥	⑦	□E1.1
E1.2 爱挑剔的，好争论	①	②	③	④	⑤	⑥	⑦	□E1.2
E1.3 可靠的，自律的	①	②	③	④	⑤	⑥	⑦	□E1.3
E1.4 焦虑的，易心烦的	①	②	③	④	⑤	⑥	⑦	□E1.4
E1.5 容易接纳新事物的，能容忍复杂事物的	①	②	③	④	⑤	⑥	⑦	□E1.5
E1.6 保守的，安静的	①	②	③	④	⑤	⑥	⑦	□E1.6
E1.7 有同情心的，热心的	①	②	③	④	⑤	⑥	⑦	□E1.7
E1.8 条理不清的，粗心的	①	②	③	④	⑤	⑥	⑦	□E1.8
E1.9 平静的，情绪稳定的	①	②	③	④	⑤	⑥	⑦	□E1.9
E1.10 传统的，枯燥的	①	②	③	④	⑤	⑥	⑦	□E1.10

C13 以下对死亡的态度您同意吗？同意程度如何？

	完全不同意	比较不同意	有点不同意	说不清	有点同意	比较同意	完全同意	
E2.1 死亡意味着一切的结束，令我害怕。	①	②	③	④	⑤	⑥	⑦	□E2.1
E2.2 我总是试着不要想到死亡。	①	②	③	④	⑤	⑥	⑦	□E2.2
E2.3 我盼望死后能和我所爱的人团聚。	①	②	③	④	⑤	⑥	⑦	□E2.3
E2.4 死亡只是生命过程的一部分。	①	②	③	④	⑤	⑥	⑦	□E2.4
E2.5 我视死亡为现世痛苦的解脱。	①	②	③	④	⑤	⑥	⑦	□E2.5

D 医疗与患病

D 1 您是否患有下列疾病？如果全部没有请在D1框内打 √ ☐D1no

**疾病名称**	是否有病<br>有，在○打√ 没有，不填
D 1.1 高血压	○D 1.1
D 1.2 糖尿病	○D 1.2
D 1.3 心脏病	○D 1.3
D 1.4 中风及脑血管疾病	○D 1.4
D 1.5 支气管炎，肺气肿，哮喘病或肺炎	○D 1.5
D 1.6 肺结核	○D 1.6
D 1.7 白内障	○D 1.7
D 1.8 慢性肾炎	○D 1.8
D 1.9 癌症	○D 1.9
D 1.10 颈腰椎疾病	○D 1.10
D 1.11 胃肠疾病	○D 1.11
D 1.12 帕金森氏病	○D 1.12
D 1.13 跌倒	○D 1.13
D 1.14 关节炎	○D 1.14
D 1.15 痴呆	○D 1.15
D 1.16 肢体残疾	○D 1.16
D 1.17 代谢紊乱（血糖、血脂、血尿酸水平升高）	○D 1.17
D 1.18 其他，请说明	○D 1.18

D2 您过去一年到医院或诊所看过病吗？ 1=无 2=______次 D2a ☐D2

D3 您最近两年是否住过院？ 1=无 2=______次 D3a ☐D3

D4 您现在经常服用的药物有几种? 1=无 2=______种 D4a ☐D4

D5 您现在参加了以下哪些医疗保险？（多选题） ☐D5

1=基本医疗保险（职工基本医疗保险、城镇居民基本医疗保险、新型农村合作医疗、城乡居民基本医疗保险） 2=城乡居民大病医疗保险 3=公费医疗

4=商业医疗保险 5=都没有

D6 过去一年您自费共花了________元医疗费？（仅个人自费部分，含看病、住院、买药）

☐☐☐☐☐D6

D7 您觉得去医院(诊所)看病方便吗？ ☐D7

1=非常方便 2=比较方便 3=一般 4=不太方便 5=很不方便

D8 当您感冒时通常会如何处理？ ☐D8

1=马上就医 2=通过网上或其他方式询医问药 3=自己买药或自我治疗

4=观察几天，暂不处理 5=完全不处理，等待自愈 6=其他_________D8oth

D9 您多长时间做一次体检？ ☐D9

1=不做 2=每年一次 3=每两年一次 4=不定期

D10 您 18 岁以后是否打过疫苗？ ☐D10

1=没有 2=打过 3=记不清

D11 您对健康怎样理解？ ☐D11

1=健康就是无病无伤无残的状态

2=医院和医生是维护健康的主要依靠

3=维护好健康是一种自我责任

4=养生保健是维护健康的主要方式

D12 请选择以下您认同的情况和认同程度。

	非常不同意	不同意	说不清	同意	非常同意	
E3.1 在经历艰难困苦后，我能很快地恢复过来。	1	2	3	4	5	☐E3.1
E4.1 年纪越大就越没用。	1	2	3	4	5	☐E4.1
E4.2 老年和年轻时一样幸福。	1	2	3	4	5	☐E4.2
E4.3 在当今社会，老年人的价值没有得到充分发挥。	1	2	3	4	5	☐E4.3
E4.4 老年人是社会的负担。	1	2	3	4	5	☐E4.4

D13 请选择以下符合您的情况。

	回答					
E5.1 当我遇到困难，总有可信赖的朋友帮助我。	①不符合	②较不符合	③一般	④较符合	⑤符合	☐E5.1
E5.2 我善解人意。	①不符合	②较不符合	③一般	④较符合	⑤符合	☐E5.2
E5.3 我的心情通常很轻松、愉快。	①不符合	②较不符合	③一般	④较符合	⑤符合	☐E5.3
E5.4 我对新事物感兴趣。	①不符合	②较不符合	③一般	④较符合	⑤符合	☐E5.4

E 心理部分

E6 请确定过去的一周中最符合您的实际情况

请依次对下列陈述做出回答	不符合	有些符合	符合	非常符合	
E6.1 我觉得很难让自己平静下来。	0	1	2	3	☐E6.1
E6.2 我感到口干舌燥。	0	1	2	3	☐E6.2
E6.3 我好像不再有愉快的感觉。	0	1	2	3	☐E6.3
E6.4 我感到呼吸困难（比如在没有剧烈运动的情况下，也会感到呼吸急促，气喘）。	0	1	2	3	☐E6.4
E6.5 我觉得很难找到动力去学习。	0	1	2	3	☐E6.5
E6.6 我对事情往往过度反应。	0	1	2	3	☐E6.6
E6.7 我感到颤抖（比如双手发抖）。	0	1	2	3	☐E6.7
E6.8 我觉得自己消耗了很多精力。	0	1	2	3	☐E6.8
E6.9 我忧虑一些会让自己恐慌或出丑的场合。	0	1	2	3	☐E6.9
E6.10 我觉得自己对将来没有什么可盼望的。	0	1	2	3	☐E6.10
E6.11 我感到忐忑不安。	0	1	2	3	☐E6.11
E6.12 我觉得很难放松自己。	0	1	2	3	☐E6.12
E6.13 我感到忧郁、沮丧。	0	1	2	3	☐E6.13
E6.14 我无法容忍任何阻碍我继续学习的事情。	0	1	2	3	☐E6.14
E6.15 我觉得自己快要得恐慌症了（没有原因地感到恐惧，惊慌）。	0	1	2	3	☐E6.15
E6.16 我对任何事都没有热情。	0	1	2	3	☐E6.16
E6.17 我觉得自己不怎么配做人。	0	1	2	3	☐E6.17
E6.18 我觉得自己很容易被触怒。	0	1	2	3	☐E6.18
E6.19 在没有运动或劳动时，我也觉得自己的心跳不正常（比如心跳过快）。	0	1	2	3	☐E6.19
E6.20 我无缘无故地感到害怕。	0	1	2	3	☐E6.20
E6.21 我感到生命毫无意义。	0	1	2	3	☐E6.21

F 经济状况

F1 您最主要的两项生活来源是什么？ □F1a □F1b

第一位________F1a　　第二位__________F1b

1=劳动所得或工资　2=离/退休金/养老金　3=学生贷款/助学金　4=配偶/伴侣收入　5=子女资助　6=父母资助　7=其他亲属资助　8= 政府/社团的救助/补贴/资助　9=积蓄或财产性收入（利息、租金等）　10=其他_____________F1oth

F2 您去年个人的总收入是______万元 F2a；您去年平均每月花了___________元 F2b。

F3 您的经济状况怎么样？ □F3

1=非常宽裕　2=比较宽裕　3=基本够用　4=比较困难　5=非常困难

F4 目前您参加了哪些养老保险项目？（多选） □F4

1=职工基本养老保险　2=机关事业单位养老保险　3=城乡居民基本养老保险（城镇居民社会养老保险和新型农村社会养老保险）　4=企业年金/职业年金　5=商业养老保险　6=都没有

F5 您有产权属于自己的房子吗？ 1=有　2=无 □F5

G 社会网络和支持

G1 您目前在世的亲属有以下哪些？（多选） □G1

1=父亲　2=母亲　3=兄弟姐妹，____人 G1a　4=配偶　5=儿子，____人 G1b　6=女儿，____人 G1c　7=（外）祖父母　8= （外）孙子女　9=配偶的父母　10=配偶的兄弟姐妹　11=曾（外）祖父母　12=曾（外）孙子女　13=其他亲戚　14=都没有

G2 以下哪些人和您同吃同住（多选）： □G2

1=父母　2=兄弟姐妹　3=配偶/未婚伴侣　4=子女　5=媳/婿　6=配偶的父母　7=祖父母　8=孙辈子女　9=孙辈子女的配偶　10=兄弟姐妹的配偶　11=其他亲属　12=其他非亲属　13=独居

G3 您和家庭成员交流得多吗？

	经常	一般	很少	不适用	
G3.1 与父亲	1	2	3	4	□G3.1
G3.2 与母亲	1	2	3	4	□G3.2
G3.3 与子女	1	2	3	4	□G3.3
G3.4 与兄弟姐妹	1	2	3	4	□G3.4

G4 您和您配偶的关系如何？ □G4

1=非常满意 2=比较满意 3=一般 4=不太满意 5=非常不满意 6=不适用

G5 您对自己工作单位的人际关系满意吗？

	非常满意	比较满意	一般	不太满意	非常不满意	不适用	
G5.1 与上级	1	2	3	4	5	6	□G5.1
G5.2 与同级	1	2	3	4	5	6	□G5.2
G5.3 与下属	1	2	3	4	5	6	□G5.3

G6 当您遇到下列情况时最先想要找谁帮忙?

	近亲属	其他亲属	朋友 / 同事	社会机构	没人可找	谁也不找	
G6.1 心情不好想聊聊	1	2	3	4	5	6	□G6.1
G6.2 有大事要商量	1	2	3	4	5	6	□G6.2
G6.3 需要借一大笔钱	1	2	3	4	5	6	□G6.3
G6.4 生病严重需照顾	1	2	3	4	5	6	□G6.4

G7 常常有人找您商量大事吗？ □G7

1=经常 2=不太多 3=很少 4=几乎没有

G8 日常一天里您大概和多少人接触？（包括点头、打招呼、交谈、打电话、写信、网上联系都算在一起） □G8

1=0 人 2=1-4 人 3=5-9 人 4=10-19 人 5=20-49 人 6=50 人及以上

G9 您手机、电话通讯录上有多少人？________人 G9a；其中关系密切的朋友_______人 G9b。

G10 您同意社会上绝大多数人是可以信任的吗？ □G10

1=非常同意 2=比较同意 3=不太同意 4=非常不同意 5=不好说

G11 评价一下您与亲属、朋友交往中提供帮助和支持的情况？

	我得到帮助更多	我提供帮助更多	大致相当	说不清	
G11.1 近亲属	1	2	3	4	☐G11.1
G11.2 其他亲属	1	2	3	4	☐G11.2
G11.3 朋友/同事	1	2	3	4	☐G11.3

G12 您对以下社会服务的需求和使用情况。

服务项目	是否需要	是否用过	是否满意
	1=是(跳问同行是否用过） 0=否	1=是(跳问同行是否满意) 0=否	1=满意 2= 一般 3=不满意 4=无法回答
G12.1 托育服务	[____]G12.1a	[____]G12.1b	[____]G12.1c
G12.2 学前教育	[____]G12.2a	[____]G12.2b	[____]G12.2c
G12.3 就业培训	[____]G12.3a	[____]G12.3b	[____]G12.3c
G12.4 健康教育	[____]G12.4a	[____]G12.4b	[____]G12.4c
G12.5 心理咨询	[____]G12.5a	[____]G12.5b	[____]G12.5c
G12.6 法律援助	[____]G12.6a	[____]G12.6b	[____]G12.6c
G12.7 家政服务	[____]G12.7a	[____]G12.7b	[____]G12.7c
G12.8 上门看病	[____]G12.8a	[____]G12.8b	[____]G12.8c
G12.9 康复/护理	[____]G12.9a	[____]G12.9b	[____]G12.9c
G12.10 社区助餐	[____]G12.10a	[____]G12.10b	[____]G12.10c
G12.11 托老所	[____]G12.11a	[____]G12.11b	[____]G12.11c

H 居住环境

H1 您现在住房在什么位置？ □H1

1=市/县城的中心城区　2=市/县城的边缘城区　3=市/县城的城乡结合部

4=市/县城区以外的镇　5=农村　6=其他________H1oth

H2 您现在居住的社区类型属于下列哪一种？ □H2

1=未经改造的街坊型老住区　2=单位家属区　3=保障房小区　4=普通商品房小区

5=别墅区或高级住宅区　6=村改居、村居合并或“城中村”　7=农村自建房

8=其他________H2oth

H3 您的住房属于哪种类型？ □H3

1=无电梯楼房____层 H3a　2=有电梯楼房____层 H3b　3=平房　4=其他_____H3oth

H4 您现在住的房子建筑面积 ________平方米 H4a；住了________年 H4b。

H5 您对目前的住房状况满意吗？ □H5

1=非常满意（跳问 H7）　2=比较满意　3=一般　4=不太满意　5=非常不满意

H6 您对这个房子哪些方面不太满意？（多选） □H6

1=没电梯　2=没有无障碍坡道　3=面积小　4=通风采光不好　5=噪音大

6=地面滑/有高差　7=洗澡不方便　8=上厕所不方便　9=做饭不方便

10=没有紧急呼叫设备　11=上网信号不好

H7 您家步行十五分钟范围内有哪些设施？（多选）

H7.1 生活类	1=公交车站/地铁站 2=商场/超市/菜市场 3=餐馆/食堂 4=公厕	□H7.1
H7.2 文体类	1=公园/绿地　2=运动场馆/场地　3=老年活动中心/站	□H7.2
H7.3 医养类	1=医院/诊所　2=药店　3=养老院/老年日间照料中心	□H7.3
H7.4 教育类	1=幼儿园　2=小学/中学	□H7.4
H7.5 邮电金融类	1=银行　2=邮局　3=快递送达设施	□H7.5

H8 您对所居住社区环境最不满意的有下列哪些方面？(多选题) □H8

1=绿化　2=卫生　3=治安　4=道路/照明　5=邻里关系　6=物业服务

7=活动场地　8=社区活动　9=生活不方便　10=其他________H8oth　11=都没有

H9 总的来说,您对所居住社区环境满意吗？ □H9

1=非常满意　2=比较满意　3=一般　4=不太满意　5=非常不满

H10 请调查者填写确认： 1=A-H 部分已完成　2=未完成　3=作废　□H10

I 日常生活能力 （I-J 部分适用于 50 岁及以上年龄的人群）

I1 您日常生活中会有以下问题吗？

项 目	可自理	轻度依赖	中度依赖	不能自理	判断评分
I1.1 修饰	独立完成<br>1	—<br>2	在协助下和适当的时间内，能完成部分梳洗活动<br>3	完全需要帮助<br>4	□I1.1
I1.2 洗澡	独立完成<br>1	洗澡需要协助<br>2	洗澡需要协助<br>3	完全需要帮助<br>4	□I1.2
I1.3 用厕	不需协助，可自控<br>1	偶尔失禁，但基本上能如厕或使用便具<br>2	经常失禁，在很多提示和协助下尚能如厕或使用便具<br>3	完全失禁、完全需要帮助<br>4	□I1.3
I1.4 穿衣	独立完成<br>1	—<br>2	需要协助，在适当的时间内完成部分穿衣<br>3	完全需要帮助<br>4	□I1.4
I1.5 吃饭	独立完成<br>1	—<br>2	需要协助，如切碎，搅拌食物等<br>3	完全需要帮助<br>4	□I1.5
I1.6 室内行走	独立完成所有活动<br>1	借助较小的外力或辅助装置能完成站立、行走、上下楼梯等<br>2	借助较大的外力才能完成站立、行走、不能上下楼梯<br>3	卧床不起，活动完全需要帮助<br>4	□I1.6

I2 您处理以下事物需要帮助吗？

项 目	1 不需要帮助	2 需要一些帮助	3 完全做不了	
I2.1 上街购物	1 不需要帮助	2 需要一些帮助	3 完全做不了	□I2.1
I2.2 家务	1 不需要帮助	2 需要一些帮助	3 完全做不了	□I2.2
I2.3 交通	1 不需要帮助	2 需要一些帮助	3 完全做不了	□I2.3
I2.4 使用手机、电话能力	1 不需要帮助	2 需要一些帮助	3 完全做不了	□I2.4
I2.5 服用药物	1 不需要帮助	2 需要一些帮助	3 完全做不了	□I2.5
I2.6 处理财务	1 不需要帮助	2 需要一些帮助	3 完全做不了	□I2.6

I2.7 电子支付	1 不需要帮助	2 需要一些帮助	3 完全做不了	☐I2.7

J 认知评估

J1.1 注意（每个词 1 分，满分 5 分） 得分：_____/5 ☐J1.1

J1.1a 指导语："现在做一个记忆力检查。我会念出几个词，请认真听。我念完后，请重复一遍记住，过一会儿我还要问您。" ☐J1.1a

1=鼻子	2=丝绸	3=寺庙	4=菊花	5=红色

J1.2 执行功能/言语（每个动物 0.5 分，满分 9 分）得分：_____/9 ☐J1.2

指导语："接下来，请告诉我尽可能多的动物的名字。您可以说想起来的任何动物。时间是 1 分钟。请准备好[稍作停顿]。现在，请开始。[同时计时 1 分钟]。停。"

J1.2a 流畅性：在 1 分钟内尽可能多的说出动物的名字。正确回答个数 [ ] ☐J1.2a

J1.3 定向（每题 1 分，满分 6 分）得分：_____/6 ☐J1.3

J1.3a 指导语："告诉我今天的日期"。如果受试者没有说出完整的答案，则提示说："告诉我今天是哪一年，哪一月，几号，星期几。" 然后说： "现在，告诉我这个地方的名字，和这个城市的名字。"

☐J1.3a

日[ ]　月[ ]　年[ ]　星期几[ ]　地点[ ]　城市[ ]

J1.4 记忆（每个词语最高 2 分，满分 10 分）得分：_____/10 ☐J1.4

J1.4a 指导语：无提示回忆——"之前，我曾经给您读过几个词，让您记住。现在把您还能想起来的词都告诉我。" J1.4b 对于没有回忆出的词语，给出分类提示。如果受试者在分类提示下仍然没有回忆出来，则给多选提示。例如："您觉得这些词里，哪一个是我刚才读过的？[清晰读出：]鼻子，手掌，面孔。"无提示下，回忆正确的词语，每个 2 分。分类或多选提示下，回忆正确的词语，每个 1 分。 ☐J1.4a

☐J1.4b

	1=鼻子	2=丝绸	3=寺庙	4=菊花	5=红色
无提示					
分类提示	身体的一部分	一种纺织品	一种建筑物	一种花	一种颜色
多选提示	鼻子，面孔，手掌	尼龙，棉布，丝绸	寺庙，学校，医院	玫瑰，菊花，郁金香	红色，蓝色，绿色

总得分（满分 30 分）：__________ □J1

J2 请调查者填写确认： 1=A-J 部分已完成 2=未完成 3=作废 □J2

K 体格与体能测试

K1 体重 ______________ 公斤 □□□K1

K2 身高 ______________ 厘米 □□□K2

K3 腰围 ______________ 厘米 □□□K3

K4 臀围 ______________ 厘米 □□□K4

K5 血压 ______/ ______ mm Hg □□□K5a/□□□K5b

K6 握力（两次取最大值） 1=无法完成 2=完成 □K6

K6.1 左手_____公斤/ K6.2 右手____公斤 □□K6.1 /□□K6.2

K6.3 惯用手：1=左手 2=右手 □K6.3

K7 步速测定 日常速度走 6 米时间 _________ 秒 K7a □□. □K7a

6 米步数 _________ 步 K7b □ □K7b

K7.1 使用行走辅具： 1=否 2=是 □K7.1

K7.2 仅在 75 岁以下年龄组中进行：

最快速度走 6 米时间__________ 秒 K7.2a □. □□□. □K7.2a

最快速度 6 米步数 __________ 步 K7.2b □□. □K7.2b

K8 起立行走试验：要求受试者从有扶手（65cm）的椅子,座高 46cm 的椅子上站起，走 3 米，转身走回来，坐下，可用拐杖或助行器，但不能搀扶。 □K8

1=无法完成 2=拒绝 3=完成

K8.1 所用时间：_________秒 □□. □□K8.1

K9 三姿站立测评

K9.1 睁眼双脚并拢站立 10 秒，完成___________秒 □K9.1

K9.2 睁眼双脚错位站立 10 秒，完成___________秒 □K9.2

K9.3 睁眼双脚前后一线站立 10 秒，完成___________秒 □K9.3

K10 闭眼双脚提踵站立 完成 _________ 秒 □□K10

K11 起坐时间测试: 从椅子上站起，让其尽快地起立 5 次。 □K11

1=完成 2=部分完成 3=没完成

完成 _________秒 K11a □□. □□K11a

K12 光反应时测试：

手: _______ 秒 K12a □□. □□K12a

脚: _______ 秒 K12b □□. □□K12b

L 辅助检测

（仅在调查对象为体检对象，有同样常规体检项目时填写相关信息）

L1 心电图： □L1

1=未检测（跳转 L2） 2=检测

L1.1 1=未见异常 2=有异常发现 □L1.1

L2 眼底镜检： □L2

1=未检测(跳转 L3） 2=检测 □L2

L2.1 眼底动脉硬化： 1= 有 2= 无 □L2.1

L3 腹部超声：

1=未检测(跳转 L4） 2=检测 □L3

L3.1 1=未见异常 2=有异常发现 □L3.1

L3.2 设备型号______________________________ □L3.2

L4 BIA □L4

1＝未检测（跳转 L5） 2＝检测

L4.1 检测序号______________________________ □L4.1

L5 DAXE

1＝未检测 （跳转 L6） 2＝检测 □L5

L5.1 检测序号______________________________ □L5.1

L6 脑部核磁

1＝未检测（跳转 M） 2＝检测 □L6

L6.1 检测序号______________________________ □L6.1

M 实验室检查

M1 血常规： 1= 已检查 2=未检查 □M1

M1a 白血球 WBC _________ $*10^9/L$ □□ □□. □M1a

M1b 红血球 RBC _________ $*10^{12}/L$ □□ □□. □M1b

M1c 血小板 PT _________ $*10^9/L$ □□ □□. □M1c

M1d 血色素 Hb _________ g/L □□ □□. □M1d

M1e 网织红细胞 _________ % □□ □□. □M1e

M2 尿常规： 1= 已检查 2=未检查 □M2

M2a 酸碱度 PH __________ □□ □□. □M2a

M2b 尿比重 SG ___________ □□ □□. □M2b

M2c 尿蛋白 Pro ___________*g/L □□ □□. □M2c

M2d 葡萄糖 _____________mmol/L □ □□. □□M2d

M2e 酮体 _____________mmol/L □ □□. □□M2e

M2f 胆红素 _____________μmol/L □ □□. □□M2f

M2g 尿胆原 _____________μmol/L □ □□. □□M2g

M2h 亚硝酸盐 _____________ □ □□. □□M2h

M2i 尿白细胞 WBC __________个/HP □□ □□. □M2i

M2j 尿红细胞 _____________个/HP □ □□. □□M2j

M3 便常规： 1= 已检查 2=未检查 □M3

M3a 潜血 1= 阳性（+） 2= 阴性（-） □M3a

M4 血生化： 1= 已检查 2=未检查 □M4

M5 空腹血糖 GLU _________ mmol/L □ □□. □□M5

M6 糖化血红蛋白 HbA1c_______% □ □□. □□M6

M7 糖化血清蛋白 _____________μmol/L □ □□. □□M7

M8 甘油三脂 TG _________ mmol/L □ □□. □□M8

M9 总胆固醇 TC _________ mmol/L □ □□. □□M9

M10 高密度脂蛋白 HDL-C_______mmol/L □ □□. □□M10

M11 低密度脂蛋白 LDL-C_______mmol/L □ □□. □□M11

M12 血清肌酐 Cr___________μmol/L □ □□. □□M12

M13 血尿素氮 BUN_________mmol/L □ □□. □□M13

M14 血尿酸 UA___________μmol/L □ □□. □□M14

M15 丙氨酸氨基转移酶 AHT _________U/L □ □□. □□M15

M16 门冬氨酸氨基转移酶 AST_________ U/L □ □□. □□M16

M17 总胆红素 TBiL_________μmol/L □ □□. □□M17

M18 总蛋白 _____________g/L □ □□. □□M18

M19 白蛋白 _____________g/L □ □□. □□M19

M20 前白蛋白 _____________mg/dL □ □□. □□M20

M21 超敏 C-反应蛋白 _____________mg/L □ □□. □□M21

M22 β2 微球蛋白 _____________mg/L □ □□. □□M22

M23 γ-谷氨酰转肽酶 _____________U/L □ □□. □□M23

M24 总胆汁酸 _____________μmol/L □ □□. □□M24

M25 血清乳酸脱氢酶 _____________U/L □ □□. □□M25

M26 肌酸激酶 _____________U/L □ □□. □□M26

M27 胆碱酯酶 ____________U/L □ □□. □□M27

M28 碱性磷酸酶 ____________U/L □ □□. □□M28

M29 钾 __________mmol/L □ □□. □□M29

M30 钠 __________mmol/L □ □□. □□M30

M31 氯 __________mmol/L □ □□. □□M31

M32 钙 __________mmol/L □ □□. □□M32

M33 无机磷 __________mmol/L □ □□. □□M33

M34 血清胱蛋白抑制剂 C __________mg/L □ □□. □□M34

M35 血清同型半胱氨酸 __________μmol/L □ □□. □□M35

M36 超氧化物歧化酶 __________U/L □ □□. □□M36

M37 血清叶酸 __________ng/mL □ □□. □□M37

M38 游离脂肪酸 __________mmol/L □ □□. □□M38

M39 雌二醇 __________pg/mL □ □□. □□M39

M40 总睾酮 __________ng/mL □ □□. □□M40

M41 胰岛素 __________μU/mL □ □□. □□M41

M42 胰岛素样生长因子 __________ng/mL □ □□. □□M42

M43 免疫球蛋白 M __________mg/dl □ □□. □□M43

M44 甲胎蛋白 __________ng/mL □ □□. □□M44

M45 癌胚抗原 __________ng/mL □ □□. □□M45

M46 鳞状上皮细胞癌相关抗原_________ng/mL □ □□. □□M46

M47 D-3 羟基丁酸 __________mmol/L □ □□. □□M47
