# Supplementary material for: Prevalence of sarcopenia was higher in women than in men: a cross-sectional study from a rural area in eastern China
Source: PeerJ. 2022 Aug 2;10:e13678. doi: 10.7717/peerj.13678 (PMC9354735; doi:10.7717/peerj.13678)
Supplement: Supplemental Information 3 — Most of the original questionnaires were not related to the manuscript, so we translated only those parts of the questionnaire related to our manuscript. [file peerj-10-13678-s003.docx]

National key R & D program

(No. 2018YFC2000301)

Baseline Questionnaire on the Characteristics and Laws of Health Status Changes during Aging in China

**Enrollment Criteria: whether it meets the enrollment criteria □ yes = 1 □ no = 2**

1) Local residents of appropriate age who have relatively stable work and residence in the local area;

2) No acute or chronic infectious diseases;3) Non progressive fatal disease;

4) No mental illness;5) No history of alcohol or drug abuse;

6) No criminal record;

**Exclusion Criteria: whether it meets the exclusion criteria □ yes = 1 □ no = 2**

1) All personnel with possible risks of migration and mobilization.

2) Those who have any physical or mental function problems and cannot complete this survey

**Survey location:** □ province (city) □ County

**Subject No.: □□□□□□□□□□□**

**Date: □□□□/□□/□□**

**Source of Respondents:** 1 = urban community; 2 = urban unit; 3 = town; 4 = rural area; 5 = hospital (physical examination center, outpatient service); 6 = other

**Investigator code of this questionnaire: □□□□**

March 2019

**A Basic Information**

**A1 Name:___________**

**A2 Gender:** 1 = male 2 = female

**A3 Age:** ______years old

**A4 Highest education completed:** 1 = illiterate 2 = primary school 3 = junior school 4 = high school 5 = junior college 6 = undergraduate 7 = Master 8 = doctoral

**A5** S**ource of information:** 1 = myself 2 = family 3 = nanny 4 = others

**B lifestyle**

**B1 Do you often take part in physical exercise?**

| mode | Example type | Times / week | Minutes / time |
| --- | --- | --- | --- |
| aerobic exercise | 1 = walking 2 = running 3 = swimming 4 = square dancing |  |  |
| Balance movement | 1 = Taijiquan 2 =_______ |  |  |
| Strength sports | 1 = instrument 2 =_______ |  |  |
| flexible sports | 1 = Yoga 2 =_______ |  |  |
| ball games | 1 = Table Tennis 2 = badminton 3 =_______ |  |  |

1 = no (skip) 2 = yes, please answer the following questions (multiple choices)

**B2 your eating habits**

staple food habit: 1 = white rice 2 = cooked wheaten food 3 = coarse food grain 4 = Both rice and noodles

| Meat | 1 = no | 2=occasionally | 3 =often | 4 = every day |
| --- | --- | --- | --- | --- |
| Egg | 1 = no | 2=occasionally | 3 =often | 4 = every day |
| Fish | 1 = no | 2=occasionally | 3 =often | 4 = every day |
| Legumes | 1 = no | 2=occasionally | 3 =often | 4 = every day |
| milk or dairy products | 1 = no | 2=occasionally | 3 =often | 4 = every day |
| vegetables | 1 = no | 2=occasionally | 3 =often | 4 = every day |
| fruit | 1 = no | 2=occasionally | 3 =often | 4 = every day |

**B3 do you drink?**

1 = don't drink alcohol 2 = drink often now 3 = drink often in the past and quit

**B4 do you smoke?**

1 = never smoke 2 = smoke now (> 5 cigarettes / day) 3 = smoke occasionally 4 = smoke in the past and quit (no smoking for more than 1 month)

**C Medical Treatment and Illness**

| Disease | Yes, tick √; no, don't fill in |
| --- | --- |
| hypertension |  |
| diabetes mellitus |  |
| heart disease |  |
| stroke and cerebrovascular diseases |  |
| bronchitis, emphysema, asthma or pneumonia |  |
| tuberculosis |  |
| cataract |  |
| chronic nephritis |  |
| cancer |  |
| cervical and lumbar diseases |  |
| gastrointestinal diseases |  |
| Parkinson's disease |  |
| falls |  |
| arthritis |  |
| dementia |  |
| physical disability |  |
| metabolic disorder (elevated levels of blood glucose, blood lipid and blood uric acid) |  |
| others, please specify |  |

**Do you have the following diseases?**
